# Neutrophil-Derived Proteases in Lung Inflammation: Old Players and New Prospects

**DOI:** 10.3390/ijms25105492

**Published:** 2024-05-17

**Authors:** Coby J. Cheetham, Michael C. McKelvey, Daniel F. McAuley, Clifford C. Taggart

**Affiliations:** 1 Airway Innate Immunity Research (AiiR) Group, Wellcome-Wolfson Institute for Experimental Medicine and Wellcome-Wolfson Institute for Experimental Medicine, School of Medicine, Dentistry and Biomedical Sciences, Queen’s University Belfast, Belfast BT9 7BL, UK; ccheetham01@qub.ac.uk (C.J.C.); m.mckelvey@qub.ac.uk (M.C.M.); 2 Wellcome-Wolfson Institute for Experimental Medicine, Queen’s University Belfast, 97 Lisburn Road, Belfast BT9 7BL, UK; d.f.mcauley@qub.ac.uk

**Keywords:** lung disease, neutrophil, proteases, antiproteases, inflammation

## Abstract

Neutrophil-derived proteases are critical to the pathology of many inflammatory lung diseases, both chronic and acute. These abundant enzymes play roles in key neutrophil functions, such as neutrophil extracellular trap formation and reactive oxygen species release. They may also be released, inducing tissue damage and loss of tissue function. Historically, the neutrophil serine proteases (NSPs) have been the main subject of neutrophil protease research. Despite highly promising cell-based and animal model work, clinical trials involving the inhibition of NSPs have shown mixed results in lung disease patients. As such, the cutting edge of neutrophil-derived protease research has shifted to proteases that have had little-to-no research in neutrophils to date. These include the cysteine and serine cathepsins, the metzincins and the calpains, among others. This review aims to outline the previous work carried out on NSPs, including the shortcomings of some of the inhibitor-orientated clinical trials. Our growing understanding of other proteases involved in neutrophil function and neutrophilic lung inflammation will then be discussed. Additionally, the potential of targeting these more obscure neutrophil proteases will be highlighted, as they may represent new targets for inhibitor-based treatments of neutrophil-mediated lung inflammation.

## 1. Introduction

Inflammatory lung diseases, both chronic and acute, are major causes of morbidity and mortality worldwide, and consequently put huge pressure on healthcare systems and hospitals [1,2,3,4,5,6]. As the most abundant circulating leukocyte [7,8], neutrophils have a critical role in inflammatory responses, and neutrophilia is a key component of lung diseases such as bronchiectasis, chronic obstructive pulmonary disease (COPD), cystic fibrosis (CF) and acute respiratory distress syndrome (ARDS) [9,10,11,12,13,14,15,16]. Neutrophils arise from progenitor cells in the bone marrow, progressing from pluripotent stem cells to promyelocytes, band cells and finally mature neutrophils [17,18]. Neutrophil lineage cells can be identified by several surface markers [18], but the expression of surface markers, such as Ly6G an CD11b, changes drastically over the course of maturation [19]. Neutrophils have also been shown to exhibit different phenotypes in the context of various lung diseases. For example, in bronchiectasis, neutrophils display increased survival, decreased surface expression of CD11b, and impaired phagocytosis [9]. Additionally, neutrophils from patients with COPD seem to have altered chemotactic migration with decreased migratory accuracy [10]. As such, understanding how neutrophils function in the context of lung disease, and the mechanisms behind alterations in neutrophil phenotypes, is critical to developing better treatments.

Neutrophils have multiple unique mechanisms by which they influence infection and inflammation. For example, neutrophils can undergo a unique form of cell death known as NETosis where they release a ‘web’ of DNA called a neutrophil extracellular trap (NET) [20,21]. This process requires the decondensation of chromatin in the nucleus, as well as oxidative burst within the cell [22]. The aggregation of these structures can lead to the entrapment of pathogenic microorganisms, as well as degradation of some inflammatory mediators, facilitating the resolution of inflammation [23]. Neutrophils are also among the first cells to respond to inflammatory signals [8], particularly in the lung. Chemo-attractants such as interleukin 8 (IL-8), leukotriene B4 (LTB4), and more recently IL-17, are known to contribute to the chemotaxis and transmigration of neutrophils into the airways [24,25,26,27,28]. The levels of neutrophil-attracting chemokines have been shown to increase in acute lung inflammation [13,14], as well as in chronic lung diseases, such as CF [29,30]. Interestingly, some of these migrating neutrophils are known to remain in the lung microvasculature, forming a marginated neutrophil pool, which exists in dynamic equilibrium with the circulating neutrophil pool [31,32]. Additionally, when transmigrating into the airways of patients with CF, neutrophils have been shown to gain a unique inflammatory phenotype known as GRIM (granule releasing, immunomodulatory and metabolically active) [33,34].

Within the armament of neutrophils is an array of proteases, which act as both regulators and effectors of neutrophil function [35,36,37]. Proteases are known to contribute to the pathology of many muco-obstructive [38,39,40], and acute inflammatory lung diseases [41,42], so targeting neutrophil-derived proteases has garnered much attention as a means by which to alleviate tissue damage and loss of lung function. This review will outline the roles of neutrophil proteases in lung inflammation, both those commonly associated with neutrophils, and proteases that have less well understood roles in neutrophils. The current therapeutic options targeting neutrophilic proteases will also be highlighted, as well as their shortcomings and potential alternatives.

## 2. Old Players: Neutrophil Serine Proteases

**Figure 1:**
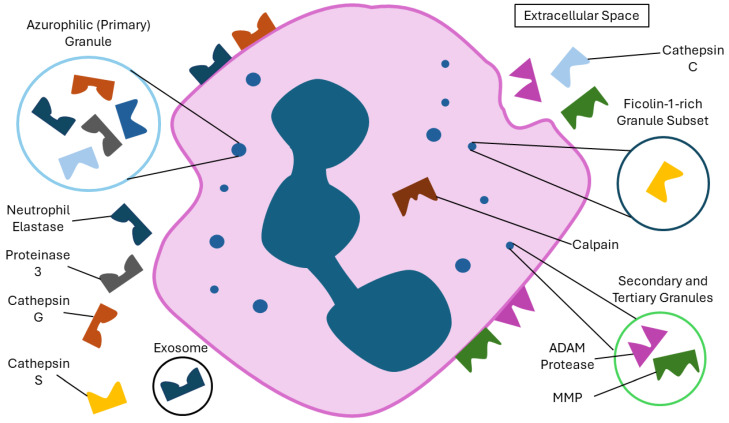
A figure illustrating the subcellular locations of various neutrophil-derived proteases. Many proteases are stored in neutrophil granules and subsequently released into the extracellular environment during degranulation. Some are neutrophil membrane- or exosome-bound, and others, while known to be produced by neutrophils, are not known to reside in specific subcellular locations.

The neutrophil serine proteases (NSPs) are the proteases most often associated with neutrophils and neutrophilic inflammatory diseases [8]. They include neutrophil elastase (NE), cathepsin G (CTSG) and proteinase 3 (PR3), as well as the more recently discovered NSP4 [43]. They are produced during neutrophil development and are stored in the azurophilic (primary) granules (Figure 1) [44]. Interestingly, their expression levels vary throughout the maturation and life of a neutrophil [45,46], with expression first being detectable in blast cells, peaking in promyelocytic cells and waning once cells fully differentiate. As such, mature neutrophils do not synthesise NSPs, but simply store them in large amounts in the primary granules [47]. NSP proteins are initially synthesised as zymogens that are activated by cathepsin C (CTSC) via the modification of the N-terminal region of the proteins [48]. NSPs can act both intracellularly, where they degrade phagocytosed material and microorganisms, and extracellularly, where they are secreted during degranulation, and can contribute to extracellular matrix (ECM) remodelling by cleaving components, such as elastin, collagen, fibronectin and laminin [49,50]. Within the neutrophil, NSPs potentially contribute to NETosis [35,36], though their importance in this process is not fully clear due to conflicting data [21]. Dysregulation of overall NSP levels or activity contributes to the pathology of lung diseases. However, each NSP acts in a unique manner, and as such it is important to look at the function and dysregulation of each individually.

### 2.1. Neutrophil Elastase

NE was first described in 1965 by Janoff and Scherer, who found that an extract of frozen granules contained proteases, which increased vascular permeability in the skin and muscle [51]. Since then, there has been a vast amount of research into NE due to it being the most abundant NSP in the azurophilic granules [52]. NE is encoded by the gene *ELANE*, and mutations in this gene can lead to neutropaenia, likely due to a maturation arrest in the promyelocytes [53]. NE acts alongside reactive oxygen species (ROS) to mediate bacterial killing and sepsis defence, where it can cleave outer membrane proteins of bacteria, such as *Escherichia coli* [54,55,56]. As such, mutations in *ELANE* may also result in increased incidence and severity of infection.

Although initially observed in granules [51], further research has shown that NE can bind to proteoglycans on the cell surface, in a reversible and dynamic manner [57]. This mode of cell surface binding leaves the NE active site available, meaning that surface-bound NE is catalytically active. Furthermore, neutrophil exosomes are able to uptake this NE from the cell surface [58]. Critically, this surface-bound NE seems to be resistant to inhibition, and instilled NE-containing exosomes can confer destructive lung disease to mice. This highlights that NE localisation is not only more complex than originally believed, but also potentially important in the pathology and propagation of lung inflammation. The overall activity of NE in the lung not only depends on the level of NE in the neutrophils and the levels of secreted NE, but also on the levels of endogenous inhibitors in the tissues [52]. These naturally-occurring inhibitors of NSPs include the serpins [59,60,61], α1-antitrypsin (A1AT) [62], and α1-antichymotrypsin [63], as well as secretory leukocyte protease inhibitor (SLPI) and elafin [64,65,66]. Interestingly, both SLPI and elafin appear to be cleaved and inactivated by high levels of NE activity [66,67]. Therefore, in lung disease with high levels of NE, there is not only an increased level of NE but a decreased level of these endogenous inhibitors [68].

NE appears to directly contribute to mucus obstruction, which is a key component of many respiratory diseases. For example, NE upregulates the expression of secretory mucins, such as MUC5AC in the airways [69,70], altering the levels of MUC5AC mRNA and modulating the levels and activity of micro RNAs (miRNAs). NE is also suggested to induce MUC5AC secretion from the airway epithelium, via the cleavage of PAR2 and the metaplasia of goblet cells [71,72,73]. This leads to thickening of the mucus and impaired mucociliary clearance. This thickening is exacerbated by NE-mediated cleavage of cystic fibrosis transmembrane conductance regulator (CFTR) and epithelial sodium (ENaC) channels in the airways [39,74]. NE is also closely associated with pro-inflammatory signalling within the context of lung disease, where it can activate cytokines, such as IL-1α, IL-8, and IL-36δ [75,76], and produce fibrin cleavage products that promote the migration of neutrophils [52]. Despite enhancing many aspects of the immune response, NE also interferes with other aspects of immunity in the lung. Interestingly, NE can cleave antimicrobial proteins, like lactoferrin [77], and may also contribute to the defective phagocytosis seen in diseases such as CF [78]. Toll-like receptor (TLR) signalling also seems to be affected by NE, which can cleave TLRs in some pneumonias, leading to altered cytokine transcription [79]. Environmental stimuli, such as cigarette smoke and E-cigarette vapour, also induce a neutrophil-mediated inflammation, which involves NE [80,81]. Together, the years of mechanistic work regarding NE in the lung have shown a complex relationship between this protease and the immune function of the lung, which explains its involvement in a number of lung diseases [42,82,83,84,85].

### 2.2. Cathepsin G

CTSG is a serine cathepsin produced by immune cells, such as monocytes and neutrophils [86,87]. CTSG can not only be secreted from neutrophils, but can also be expressed on the cell surface, which preserves its activity in the presence of inhibitors [88]. Like NE, CTSG has several important roles in cellular and immune functions related to lung inflammation, such as cytokine processing [89,90], pathogen clearance [91,92,93], and cell-surface receptor modification [94]. However, dysregulation of CTSG activity is known to be detrimental, especially in the lung. For example, CTSG cleaves ECM proteins, such as elastin, in COPD, contributing to the loss of lung function [95,96]. CTSG is also thought to affect lung microbiome composition in patients with CF by affecting the amino acid levels in the airway environment and by cleaving important antimicrobial proteins [97,98]. There also appears to be a role for CTSG in the formation of NETs via a PAD4-mediated mechanism involving neutrophil-platelet interactions [22,99]. The association of CTSG with lung inflammation has even implicated CTSG as a potential biomarker for airway tissue damage [100]. Therefore, despite NE being the major interest regarding NSP research, CTSG also has crucial and independent roles that are worth considering in lung inflammation.

### 2.3. Proteinase 3 and NSP4

PR3 is the third classical NSP, which is known to be stored both in neutrophil azurophilic granules and in secretory vesicles [101]. The roles of PR3 within cells are varied, ranging from cytokine and antimicrobial peptide activation [102,103] to the activation of pro-apoptotic signalling pathways [104]. PR3 dysregulation is also associated with lung disease. PR3 is thought to contribute to the ECM degradation seen in emphysema [95,105] and is also detectable at high concentrations in CF sputum, where it contributes to the secretion of characteristically thick mucus [106].

The fourth NSP is the more recently discovered NSP4, which appears to be found in neutrophil granules, like the classical NSPs [43,107]. The abundance of this NSP in the granules is low, though the processing and secretion seems to be comparable to the other NSPs [107]. Apart from this, very little is known about NSP4, and its role in lung disease has been unexplored to date.

### 2.4. NSP Inhibition

Due to the involvement of the NSPs, particularly NE, in lung disease pathology, synthetic inhibitors that regulate their activity have previously been investigated in both animal models and human clinical trials. In the context of COPD, the NE inhibitor AZD9668 showed promising results in animal models utilising smoke-inhalation [108]; however, two clinical trials investigating the compound found no significant therapeutic effect for patients [109,110]. This inhibitor was also trialled in patients with bronchiectasis, and similarly variable results were observed [111], though there were slight improvements in forced expiratory volume. Similarly mixed efficacy of other NE inhibitors has also been reported in CF and non-CF bronchiectasis clinical trials [112,113]. The only NE inhibitor licenced for use is Sivelestat, which is licenced for clinical use in Japan [114]. This inhibitor has been widely investigated in the context of acute lung inflammation [115,116], and a meta-analysis of these trials showed no overall effect on mortality or length of ICU stay [117]. There are some other NE inhibitors that are still in the early trial stages, such as POL6014, which has shown effective pharmacokinetics in patients with CF [118], but has yet to be investigated regarding efficacy. A summary of these clinical studies can be found in Table 1.

Overall, patients treated with NE inhibitors seem to have a significant variability in their response to the drugs, resulting in the poor results seen in these studies. The mode of inhibition may be a key reason behind these failures, and more novel inhibition methods, such as antibody-based and miRNA-based therapeutics, are therefore being researched [122,123]. These failures of NE inhibition may also be in part due to the high day-to-day variability seen in NSP and NE activity in lung disease patients [124]. This may suggest that a more personalised approach to NSP inhibition, considering the individual NSP activity of each patient, would be much more effective. The limited success of NSP inhibition has prompted the consideration of alternative or complementary proteases that could be targeted therapeutically. Indeed, the cutting edge of protease research in lung inflammation is now moving towards proteases of which we have limited understanding in the context of neutrophil function.

## 3. New Prospects: Other Neutrophil Proteases

### 3.1. Cysteine Cathepsins

The cathepsins are a group of related proteases that were first discovered in 1968 when Drenth and colleagues described the protease papain from papayas [125]. They found that this protease had an active site consisting of an α-helical heavy chain and a short β-chain, and this form of active ‘papain-like’ domain is the link between the various cathepsin proteases [126]. The cathepsins can be organised into several families, such as the serine cathepsins, the aspartyl cathepsins, and the cysteine cathepsins [127]. Cathepsins are endopeptidases for the most part, though there are some exceptions, such as cathepsin C [48]. Cathepsins may be expressed in a ubiquitous or tissue-specific manner [127], for example, cathepsin K is expressed mainly in osteoclasts due to its involvement in bone resorption [128]. The regulation of cathepsin activity is controlled by both endogenous antiproteases as well as other factors. Some endogenous inhibitors of cathepsin activity include the cystatins, thyropins and serpins [129], and these may act either in a regulatory or emergency manner. Due to the need for reduced residues in the cathepsin active site for activity, modification of the redox environment can also lead to changes in cathepsin activity [130]. This can be done by both reactive nitrogen and oxygen species [131,132]. Additionally, some data suggest that the cleaved pro-peptides from cathepsin zymogens can act as selective inhibitors for their cognate proteases [133]. The removal of the pro-peptide seems to be promoted by some glycosaminoglycans [134], implicating molecules such as chondroitin-4-sulfate in the regulation of cathepsin activity [135,136]. Zymogens are normally processed to their mature forms in the lysosomes and are localised to these compartments via the pro-peptide [137]. The regulation of cathepsin activity is therefore complex, and highly important due to the involvement of cathepsins in many cellular functions.

Cathepsins are able to degrade and cleave ECM components, such as collagen, laminin, and fibronectin [138], and are also involved in the remodelling of tissues in the blood vessels, such as arteries [139]. Cathepsins are also important in apoptosis, where they cleave Bid and anti-apoptotic proteins [140,141], facilitating the release of cytochrome C and subsequent cell death. Cathepsins also play key roles in immunological signalling pathways, including toll-like receptor (TLR) signalling and antigen presentation [142,143], and other key cellular functions, such as autophagy, cell cycle progression and cell adhesion [144,145,146]. Their close involvement with a variety of critical cellular functions therefore explains why their dysregulation is associated with pathology; most dysregulation relating to lung diseases is aberrantly high cathepsin activity. In CF for example, the epithelial lining of the airways is acidified [147], resulting in increased cathepsin activity [148], and subsequent degradation of anti-microbial peptides [149]. This therefore implicates cathepsin activity in the tissue damage and increased incidence of infection seen in CF [150]. The upregulated cathepsin activity in COPD can also result in increased cleavage of antiproteases such as SLPI [151]. Additionally, murine models of asthma have shown a pro-inflammatory role for cathepsins in allergic airway inflammation [152,153]. These are conditions that also have a significant neutrophil component to their pathology [154,155] and understanding the roles of specific cathepsins expressed in neutrophils is therefore key to understanding neutrophil contribution to lung disease.

### 3.2. Cathepsin C

Cathepsin C (CTSC, also known as Dipeptidyl peptidase I) is a cysteine cathepsin that is expressed primarily in neutrophils and mast cells [48]. It was first described in 1948 by Gutman and Fruton [156] and was later linked to the papain-like family of cathepsin proteases after their discovery [157]. The addition of CTSC to the cathepsin family of proteases came despite its highly unique, tetrameric structure, which has not been observed for other cathepsins [158]. CTSC monomers are synthesised as zymogens in haemopoietic precursor cells [159]. These monomers contain a papain-like domain, which confers activity, a pro-peptide and an exclusion domain. The papain-like domain further consists of a heavy and a light chain. Two of these 60 kDa monomers associate to form a homodimer [160], which is then subsequently cleaved into several mature chains of CTSC [48]. This processing step results in the excision of the pro-peptide, and the remaining domains are held together by tight non-covalent interactions. Two of these processed dimers can then associate to form the active 200 kDa homo-tetramer, a process that is mediated by the exclusion domains [161]. This processing pathway is suggested to be facilitated by other cathepsins, such as cathepsins L, S, K, V and F [160], though there is little research in cell lines or animal models to confirm this. Mature CTSC plays a role in the catalytic processing of other proteases, such as the NSPs [162,163]. This happens early in the maturation process of the neutrophil [164]; as these cells mature, more CTSC is secreted into the extracellular space, as there are no new NSP mRNA transcripts being produced [45,46,159].

CTSC therefore plays a critical role in the protective inflammatory responses during infection, and some pathogens, such as *Helicobacter pylori*, are known to impair CTSC expression to promote infection [165]. Loss-of-function genetic mutations in the *CTSC* gene are also detrimental to normal immune function and cause a condition known as Papillon–Lefèvre syndrome (PLS) [166]. Many PLS-associated mutations result in impaired CTSC processing, which is detectable by the lack of heavy or light chain CTSC in urine samples [167]. The associated phenotype for this condition primarily presents as severe periodontitis and palmoplantar keratosis [166], but the levels of NSPs are also significantly reduced in PLS patients [168]. This results in reduced downstream proteolysis, such as reduced processing of LL-37 [169], which can have significant effects on inflammatory and anti-microbial responses. Neutrophil functions that rely on NSP activity are also impaired in PLS [169]. NET release and chemotaxis appear impaired in PLS neutrophils, whereas the levels of pro-inflammatory cytokines released are higher [170]. As such, PLS results in an increased susceptibility to infection in around 25% of cases [171]. Other CTSC mutations result in a more severe condition known as Haim–Munk syndrome (HMS) [172], however some evidence suggests that they are the same condition, presenting with different phenotypes [173]. Together, PLS and HMS demonstrate that CTSC is a critical component not only of neutrophil function, but cellular and immune function as a whole.

Like most proteases, CTSC activity within neutrophils must be balanced, and aberrantly high CTSC activity may also be detrimental in inflammatory diseases of the lungs. CTSC is mainly linked to NSP-mediated lung diseases, which include ARDS, CF, and COPD [80,82,85,95,106,174,175], due to its role in NSP activation. However, CTSC may also contribute to lung inflammation through alternative means. Lysosomal destabilisation is one of the key processes by which cathepsins are released into the cytosol, and lead to various forms of cell death [176]. Some interesting data suggest that CTSC is important in leucyl-L-leucine methyl ester (LLOME) mediated lysosomal destabilisation [177], leading to a rapid release of lysosomal contents. This may implicate CTSC in the action and secretion of other cathepsins, which are known to cause lung tissue damage [149,151,152]. CTSC also appears to have roles in IL-1β secretion, through a caspase-1-independent mechanism [178]. CTSC may consequently play a role in important inflammatory signalling pathways, such as the inflammasome pathway [179]. Whether this effect is directly mediated by CTSC, or if it is mediated by a downstream protease, is unknown in neutrophils, though it has been shown that mast cell chymase, another protease activated by CTSC, can cleave IL-1β in an atypical way [180]. It may therefore be the case that NSPs are involved in this alternative pathway of IL-1β activation in neutrophils.

Because of these pro-inflammatory links, targeting CTSC activity using inhibitors has been investigated in both animal models and clinical trials regarding lung disease. Most of the currently developed CTSC inhibitors are nitrile inhibitors [181,182,183,184], for example Brensocatib [185]. In rodent models, this inhibitor achieved a dose-dependent plasma exposure and a dose-dependent reduction in NSP activity in the bone marrow [186]. The success of Brensocatib in animal models led to several clinical trials in lung diseases, as well as animal models of inflammatory diseases of other organ systems [187]. While work in patients with bronchiectasis showed promising results, with a reduction in the frequency of exacerbations [188,189], work in patients with COVID-19 showed negative effects and increased mortality [190] (Table 1). Despite these issues in COVID-19, Brensocatib has been considered a success and, following promising preclinical results [191], Chalmers and colleagues have started investigating a new CTSC inhibitor BI1291583 in patients with bronchiectasis [192]. Other CTSC inhibitors have not shown strong clinical translation, for example GSK-2793660 [193], which failed due to minimal effects on NSP activity. More recent work has shown that non-peptidyl, non-covalent inhibitors of CTSC can also effectively reduce NSP activity and may be more metabolically stable than their covalent counterparts [194,195]. Overall, CTSC inhibition represents a promising option for the treatment of inflammatory lung diseases, potentially having more consistent and far-reaching effects than NSP-specific inhibitors [110,111,112,116].

### 3.3. Cathepsin S

Cathepsin S (CTSS) is a cysteine cathepsin that has somewhat restricted expression, being produced mainly in immune cells such as macrophages and neutrophils [146,196]; however, it can also be expressed in non-immune cells such as airway epithelial cells [197]. In the context of the neutrophil, though not thought to be present in primary, secondary or tertiary granules, Rørvig and colleagues have previously noted that CTSS is located in a subset of Ficolin-1-rich granules, which are likely to be among the first granules exocytosed upon stimulation [198]. Like most cathepsins, CTSS is synthesised as a zymogen, which is subsequently cleaved to its active and mature form [199]. The processing of CTSS zymogens can be facilitated by other proteases, as well as CTSS itself, in an autocatalytic process [200]. Regarding function, CTSS has the unique ability to retain its activity at neutral and even slightly alkaline pH [201], allowing CTSS to exhibit activity in the extracellular space [202]. The secretion of CTSS is usually as a result of overexpression [203] in pro-inflammatory contexts [204]. The regulation of CTSS activity both in cells and in the extracellular space is highly complex. The regulation of CTSS mRNA at a transcriptional and post-transcriptional level is reported to be mediated by molecules such as gamma interferon-inducible lysosomal thiol reductase (GLiT), human antigen R (HuR) and tristetrapolin (TTP) [205,206,207]. The activity of the CTSS protease itself is also subject to strict regulation, both through the action of endogenous inhibitors [208,209], and other mechanisms such as oxidation [210,211]. The regulation of CTSS can even be altered by certain microorganisms to facilitate their survival and spread. For example, several *Mycobacteria* species regulate CTSS through upregulating certain micro RNAs (miRNAs) and inhibiting CTSS processing through IL-10 signalling [212,213].

CTSS plays a critical role in multiple cellular functions, including the fusion of autophagosomes and lysosomes [214], and the processing of major histocompatibility complex II [143]. Interestingly, CTSS also appears to play a role in the pain signalling associated with inflammation [215]. Like most cathepsins, however, CTSS dysregulation plays a role in the pathogenesis of many diseases, including several neutrophilic lung diseases [41,154,216,217]. CTSS is also upregulated in lung inflammation induced by environmental stimuli [218,219,220]. Although the role of CTSS in tissue damage and increased airway permeability in these diseases is well-documented [119,219], the role of CTSS in neutrophils themselves is relatively unexplored to date. Some previous work looking at murine models of acute lung inflammation demonstrated that CTSS inhibition reduced neutrophil influx into the airways, but the mechanisms behind this are not understood [41]. Adding to this are recent data regarding stress responses in mice, which also found a role for CTSS in the recruitment of immune cells, such as neutrophils [120]. CTSS has previously been shown to affect migration of other cells [121], and it can activate several chemokines, such as CXCL2, via cleavage [221]. CTSS may also influence neutrophil migration and function by affecting the processing and activity of NSPs, though there is limited, conflicting evidence of its involvement [159,160,222,223]. CTSS has also recently been linked to NETosis in the context of cancer immunotherapy [224], where CTSS was found within NETs cleaving arginase to facilitate anti-tumour immunity [225,226]. Together, these data show that the role of CTSS in the inflammatory response is relatively unknown at this stage, but its critical role in many neutrophilic lung diseases highlights the need for further research in this area.

### 3.4. Other Cysteine Cathepsins

Neutrophils express other cathepsins, some at high levels and some at very low levels [227]. The involvement of many of these in neutrophil function has not been investigated to date. Cathepsin B (CTSB) has been identified as playing a role in neutrophil migration [228], by cleaving Mac-1 integrins on the endothelial surfaces of blood vessels. This gives CTSB an anti-adhesive and pro-migratory role, which has not been explored outside of this work. CTSB is known to be upregulated in various pro-inflammatory lung diseases, such as CF, COPD, and pneumonia [229,230,231], though further research is needed to investigate its roles in neutrophil function and inflammatory processes.

### 3.5. Calpains

The calpains are a family of non-lysosomal cysteine proteases, with a very broad expression profile. They exist as cytosolic inactive precursors until intracellular Ca^2+^ reaches a threshold level, triggering their activation. They are mostly associated with promoting cell death in response to Ca^2+^ through their cleavage of a wide range of proteins including cytoskeletal proteins, Bid, caspase-3, and proteins of the electron transport chain [232]. Overexuberant calpain activity has been associated with chronic pulmonary disease [233,234], and calpains of neutrophilic provenance may contribute specially to pathology. For example, calpains have previously been linked to cigarette smoke-mediated airway epithelium disruption, potentially increasing susceptibility to infection [235]. Within the neutrophil, along with their role in mediating apoptosis and necrosis [236], calpains appear to be important regulators of neutrophil movement. Calpain I (μ-calpain) inhibition promotes Rho GTPase activity, rapid random chemo-kinesis and pseudopod formation; interestingly, this inhibition reduces the directional fidelity of the neutrophil in response to IL-8 [237]. A similar phenomenon is observed when calpain I is inactivated in neutrophils treated with A1AT [238]. Thus, calpains facilitate the direct and accurate movement of neutrophils to the site of inflammation.

### 3.6. Metzincin Proteases

Proteases from the metzincin superfamily are characterised by the presence of a Zn^2+^ ion at the catalytic centre, bound by three histidine residues, which facilitates the hydrolysation of proteins. This superfamily comprises the matrix metalloprotease (MMP), a distintegrin and metalloprotease (ADAM), and ADAM proteases with a thrombospondin motif (ADAMTS) families, which have well-documented roles in lung homeostasis and pathogenesis, especially in the remodelling of the pulmonary ECM [239,240,241]. Despite the traditional association of metzincins with parenchymal and mesenchymal lung cells, expression of all three families has been reported in immune cells [242]. However, only a handful of specific metzincins are known to be expressed by neutrophils.

During homeostasis, metzincin expression tends to be low, and is upregulated during pathologies, such as COPD, ARDS, and CF [243,244,245,246]. MMP-8 (neutrophil collagenase) is perhaps the best-known neutrophil-derived metzincin, though neutrophils are also a major source of MMP-9 (gelatinase B) [247]. Stored in secondary and tertiary neutrophil granules [198], these MMPs are readily exocytosed during stimulation, and their potent proteolytic abilities facilitate the migration of neutrophils through tissues by the degradation of ECM and basement membranes. Destruction and remodelling of the ECM by proteases, derived not exclusively but significantly from neutrophils, is a key aspect in the development of many major lung diseases [240,248]. Some of the released MMPs are soluble and diffuse, while others remain membrane-bound [249], meaning that high MMP activity is retained on migrating neutrophils. Moreover, neutrophils do not appear to release the tissue inhibitor of metalloproteinase (TIMP)-1 [250,251], which may also contribute to high MMP activity in the environs of the neutrophil during stimulation and migration. MMP-8 is involved in ECM remodelling during migration, where it degrades collagen [252]. MMP-8 also cleaves non-structural substrates, such as A1AT [253], which compromises the antiprotease defence against serine proteases. Furthermore, the inflammatory ability of an array of cytokines and chemokines can be proteolytically enhanced by MMP cleavage. For instance, CXCL8 can be processed by MMP-8 and -9 [254,255], while N-acetyl proline-glycine-proline (Ac-PGP) fragments are produced by MMP-9-mediated cleavage of collagen [256]. Both cleavage processes produce highly chemotactic molecules that enhance neutrophil recruitment during lung disease. Interestingly, both CXCL8 and Ac-PGP also upregulate MMP-9 release from neutrophils, stimulating a feed-forward mechanism that propagates inflammation. Beyond MMP-8 and -9, the roles of other neutrophil-derived MMPs have not been investigated. Gene expression analysis indicates that at least two membrane-type (MT-)MMPs are expressed by primary human neutrophils (MMP-24 and -25, also known as MT5-MMP and MT6-MMP, respectively) [227]. Few clear functions have been attributed to these proteases, though MT6-MMP can cleave numerous CXC and CC chemokines, possibly indicating a role in the inflammatory process [257].

ADAM proteases are best-known for their functions in cell adhesion and the processing of ectodomains of cell surface proteins (so-called ‘sheddase’ activity), though several members of this family lack catalytic activity. Like the MT-MMPs, ADAMs contain transmembrane and cytoplasmic domains, as well as the extracellular MMP domain, though they are distinguished by the presence of extracellular EGF-like, cysteine-rich and disintegrin-like motifs [258]. Because of these various domains, ADAMs can exert complex pleiotropic effects, such as proteolysis, cytoskeletal manipulation, and cell signalling. Uniquely among the ADAMs, ADAM-8 is constitutively expressed in neutrophils and is, thus far, the most intriguing ADAM in neutrophil biology [259,260]. Recent work in the context of ARDS has highlighted that ADAM-8 plays an important role in neutrophil adhesion and transmigration via its intracellular interactions with myosin and the cytoskeleton, independent of its catalytic domain [246]. Although not a direct consequence of its protease activity, this function of ADAM-8 has important implications for neutrophil proteolysis in general, since transmigration into the lung facilitates the delivery of granule proteases to the site of inflammation [261]. ADAM-8 shares most of its extracellular substrates with other ADAMs, including pro-TNFα and CD23, indicating a degree of redundancy in protease function [258,262]. However, its resistance to endogenous control by TIMP-1 and its high expression in the context of neutrophilic disease suggest that ADAM-8 may have potential to contribute specially to disease progression [263]. The more broadly expressed ADAM-10 also seems to have a role in promoting neutrophil adhesion to fibronectin and migration in response to CXCL8, as well as contributing to the development of pulmonary oedema [264]. On the other hand, ADAM-17, with which ADAM-10 shares many functions, may play an antagonistic role by shedding pro-adhesive L-selectin from neutrophils [264,265], highlighting the importance of a finely tuned protease balance in neutrophil function. At the time of writing, the authors are unaware of any reports linking neutrophil-derived ADAMTS proteases with lung inflammation.

### 3.7. Aspartyl Proteases

Human aspartyl proteases use an aspartate-bound activated water molecule for catalysis and are relatively few in number when compared to other protease families. Most that are not found in the digestive system are stored in the lysosomes of cells, though notably cathepsin D is localised in the primary granules of neutrophils [266]. In these granules, cathepsin D appears to play a central role in promoting neutrophil apoptosis by activating caspase-8 when primary granules are released into the cytoplasm following, for instance, bacterial phagocytosis. This proapoptotic mechanism is important in the resolution of inflammation and can be blocked by protease inhibition or treatment of neutrophils with pro-survival factors, like GM-CSF [266,267]. Cathepsin D is processed by cathepsins L and B and auto-activation [268], once again illustrating the intricate interactions that occur between these enzymes to regulate activity. Optimal cathepsin D activity is facilitated by the acidic pH of lysosomes or neutrophil granules, most probably limiting its influence in the extracellular environment [269]; it is unclear whether cathepsin D secreted from neutrophils has a role in pulmonary ECM processing. Other aspartyl proteases, such as cathepsin E and legumain, do not appear to exhibit activity in neutrophils.

### 3.8. Granzymes

Granzymes are serine proteases typically released from the granules of NK and cytotoxic T lymphocytes. Their primary function is the induction of apoptosis in targeted cells, via the activation of caspases and apoptotic proteins, such as Bid. Granzymes also mediate the killing of intracellular pathogens [270]. However, the status and function of neutrophil granzymes remains controversial. Naïve spleen or blood neutrophils are not thought to express granzymes [271], though some groups report that colon tumour-associated neutrophils and neutrophils exposed to inflammatory cytokines, such as IFN-γ, or bacteria demonstrate upregulated expression of granzymes A and B [272,273,274]. The targets of neutrophil-derived extracellular granzymes have not been elucidated in the context of the lung, though it has been suggested that they may affect ECM structure and initiate anoikis in nearby cells [275].

## 4. Conclusions and Future Directions

Altogether, the future of neutrophil protease research in lung disease is at a crossroads. Despite being highly researched, the NSPs have been poor therapeutic targets, with inhibitors of NE showing mixed efficacy in human trials. These issues may be resolved by implementing a targeted, personalised approach depending on NSP levels within the airways of each patient. Future clinical trials of NSP inhibitors could therefore consider stratifying patients based on the baseline activity of individual NSPs in the lung to identify the patients most likely to benefit from inhibitor therapy. However, new tools would be required to assess NSP status at the bedside, especially in the context of critical illness, where treatment decisions must be made rapidly. Furthermore, pulmonary sampling techniques (bronchoalveolar lavage, sputum collection, etc.) would have to be carefully selected to ensure that accurate and disease-relevant readouts are obtained to inform treatment selection.

An alternative approach is to target some of the less-researched proteases in the neutrophil, which may prove to have equally or more profound roles in disease. This approach is already showing promising results, with CTSC inhibitors, such as Brensocatib, having beneficial effects not seen with NE inhibitors. The success of this drug and others should encourage future research to target the cathepsins and other less well-researched neutrophil proteases in both acute and chronic lung inflammation.

To date, there is relatively little research elucidating the role of many of these neutrophil-expressed proteases in neutrophil function, and for some proteases there is a complete lack of data [227]. It is important that the roles of these proteases in neutrophil function are investigated to determine their potential as therapeutic targets in lung inflammation. It is worth noting that, since proteases have such important homeostatic roles, there is also a case for the focused development of organ-directed (direct application to the lung), neutrophil-specific or extracellular-only inhibitors, to mitigate lingering concerns over systemic protease inhibition.

As our understanding of how neutrophil proteases process one another and participate in parallel and interacting signalling pathways continues to develop, we will increasingly appreciate which proteases represent the best targets; these may be proteases with well-known effector functions, those that act upstream as master regulators, or both. With this broader understanding, there will be the possibility of developing novel specific inhibitors, combination inhibitors or inhibitors of master regulators that control an array of downstream proteases, as is appropriate in each disease context to achieve the maximal benefit with minimal off-target effects.

## Figures and Tables

**Table 1:** Table summarising current clinical data regarding the efficacy of neutrophil elastase and cathepsin C inhibitors in inflammatory lung diseases. Abbreviations: NE—Neutrophil elastase, COPD—Chronic obstructive pulmonary disease, ARDS—acute respiratory distress syndrome, FEV1—Forced expiratory volume in 1 s, QoL—Quality of life, N/A—Not Available.

Inhibitor	COPD	Cystic Fibrosis	Bronchiectasis	ARDS
AZD9668	No significant effects on pre-bronchodilator FEV1 or QoL. No significant effect on urine desmosine [107,108].	No significant effects on sputum neutrophils, NE activity, lung function or QoL. Some statistically significant changes in IL-6 and urine desmosine [110].	No significant effects on sputum neutrophils. FEV1 improved and levels of IL-6 and IL-8 decreased significantly [109].	N/A.
Sivelestat	N/A.	N/A.	N/A.	Approved for use in Japan [112]. Meta-analysis of six randomised control trials suggests no significant effects on 28–30-day mortality or ventilation days. Some evidence suggests Sivelestat may improve P/F level and may be more effective in mild-to-moderate ARDS [114,115].
POL6014	N/A.	Good results in pre-clinical investigations and good pharmacokinetics and dynamics. Clinical investigation of efficacy not yet performed [116].	N/A.	N/A.
BAY 85-8501	N/A.	N/A.	No significant effects on FEV1. Significant reduction in NE activity; however, this had no clinical effects [111].	N/A.
Brensocatib	N/A.	N/A.	Increased time until first exacerbation and reduced overall incidence of exacerbations by around 40%. Number of severe exacerbations in treatment group were 50% lower [119,120].	Severe COVID-19 patients treated with Brensocatib had increased mortality and increased need for supplemental oxygen. Treatment did reduce NE activity in blood however [121].
